# SEVTAR—A multicenter randomized controlled trial to investigate the impact of prophylactic endoluminal placed vacuum sponge for prevention of anastomotic leakage after low rectal resections

**DOI:** 10.3389/fsurg.2022.1099549

**Published:** 2023-02-13

**Authors:** Leif Schiffmann, Matthias Becker, Leendert Develing, David Varga-Szabo, Caroline Scheidereiter-Krüger, Hubert Zirngibl, Michael Seifert, Lothar Biermann, Claudia Schlüter, Felicitas Tumczak, Arved Weimann, Boris Jansen-Winkeln, Ingo Wallstabe, Frank Schwandner, Sandra Denecke, Clemens Schafmayer, Imad Kamaleddine, Albrecht Stier, Katharina Haegele, Michael Kindler, Sabine Michling, Ernst-Wilhelm Horling, Ulrike Denzer

**Affiliations:** ^1^ Department of Visceral and General Surgery, Helios Klinikum Aue, Aue, Germany; ^2^ Department of General, Thoracic, Vascular and Transplantation Surgery, University of Rostock, Rostock, Germany; ^3^ Department of Visceral and General Surgery, Helios Weißeritztal-Kliniken GmbH—Klinikum Freital, Freital, Germany; ^4^ Department of Visceral and General Surgery, University Hospital Wuppertal, Wuppertal, Germany; ^5^ Department of Visceral and General Surgery, Helios Klinikum Erfurt, Erfurt, Germany; ^6^ Department of Visceral and General Surgery, Joseph Hospital Warendorf, Warendorf, Germany; ^7^ Department of Visceral and General Surgery, Klinikum St. Georg Leipzig, Leipzig, Germany; ^8^ Department of Gastroenterology, Klinikum St. Georg Leipzig, Leipzig, Germany; ^9^ Department of Visceral and General Surgery, Klinikum Kaufbeuren, Kaufbeuren, Germany; ^10^ Department of Gastroenterology, Marburg University Hospital, Marburg, Germany

**Keywords:** rectal cancer, anastomotic leakage, prevention, SEVTAR study, randomized trial

## Abstract

**Background:**

Low anterior resection for rectal cancer is commonly associated with a diverting stoma. In general, the stoma is closed 3 months after the initial operation. The diverting stoma reduces the rate of anastomotic leakage as well as the severeness of a potential leakage itself. Nevertheless, anastomotic leakage is still a life-threatening complication and might reduce the quality of life in the short and long term. In case of leakage, the construction can be converted into a Hartmann situation or it could be treated by endoscopic vacuum therapy or by leaving the drains. In recent years, endoscopic vacuum therapy has become the treatment of choice in many institutions. In this study, the hypothesis is to be evaluated, if a prophylactic endoscopic vacuum therapy reduces the rate of anastomotic leakage after rectal resections.

**Methods:**

A multicenter parallel group randomized controlled trial is planned in as many as possible centers in Europe. The study aims to recruit 362 analyzable patients with a resection of the rectum combined with a diverting ileostoma. The anastomosis has to be between 2 and 8 cm off the anal verge. Half of these patients receive a sponge for 5 days, and the control group is treated as usual in the participating hospitals. There will be a check for anastomotic leakage after 30 days. Primary end point is the rate of anastomotic leakages. The study will have 60% power to detect a difference of 10%, at a one-sided alpha significance level of 5%, assuming an anastomosis leakage rate of 10%–15%.

**Discussion:**

If the hypothesis proves to be true, anastomosis leakage could be reduced significantly by placing a vacuum sponge over the anastomosis for 5 days.

**Trial registration:**

The trial is registered at DRKS: DRKS00023436. It has been accredited by Onkocert of the German Society of Cancer: ST-D483. The leading Ethics Committee is the Ethics Committee of Rostock University with the registration ID A 2019–0203.

## Background

Despite all progress in improving the treatment of patients with rectal cancer with neoadjuvant therapy or total mesorectal exzision (TME), anastomosis leakage (AL) after low anterior resections remains a significant problem. The rate of insufficient anastomosis lies between 0% and 26% (1, 2). Up to 2007, the treatment of AL was limited to converting into a Hartmann situation, reoperation, or treating the patient with an intelligent regime containing the component drainage, rinsing, and diverting stoma. The diverting stoma itself led to a decrease of AL and lowered mortality (3, 4).

In 2008, Weidenhagen et al. (5) published their results of a new approach for AL by using an endoscopically placed vacuum sponge (EVT) in the insufficient cavity and a commercial system became available. This system leads to a high percentage of successful healing of AL. Until now, there is no randomized trial available comparing the results of conventional treatment vs. the EVT. Kühn et al. (6) compared their results of EVT with a historical cohort of conventionally treated AL and found a significantly higher success rate of EVT compared to conventional treatment (95.2% vs. 65.9%, *p* = 0.011). EVT was also associated with the preservation of intestinal continuity in a significantly higher percentage of patients than those undergoing conventional treatment (86.7% vs. 37.5%, *p* = 0.001).

In many hospitals in Germany and other countries, EVT for treating AL is considered the gold standard and the results are satisfying (7, 8). However, the treatment is costly and time-consuming. It might be even better if AL could be avoided in the first place. Despite attempts testing the anastomosis intraoperatively and the blood flow of the two sides of the rectum and descendens, the intraluminal placement of a vacuum sponge might lead to a significantly reduced rate of AL.

### Objectives and hypothesis

The aim of our study was, therefore, to investigate whether an intraoperatively placed sponge reduces the rate of AL compared with patients treated without sponge after rectal resection.

## Methods

The administrative and medical processes are shown in flowcharts 1 and 2.

### Design

A prospective randomized multicenter clinical superiority trial is performed with a balanced randomization. Before the inclusion of the first participant, the trial was evaluated by the Ethics Committee of Rostock University (ID: A 2019–0203). Then, the trial was registered at DRKS (DRKS00023436). It has been accredited by Onkocert of the German Society of Cancer (ST-D483).

During the course and additional centers joining the study, there were positive statements of the following ethics committees: Medical Association of Saxony, Thuringia, Westfalen-Lippe, Bavaria, Baden-Württemberg, and of the Universities of Wuppertal and Düsseldorf.

### Participants and recruitment

Individuals referred to one of the participating centers for rectal resection will be examined and screened for potential participation. Recruitment will be performed in a standard outpatient or inpatient clinic setting by a surgeon at least 24 h before the operation. Information about the study is handled over to the patient and formal consent was obtained.

### Eligibility criteria

All legally competent patients with age 18 years and older scheduled for rectal resection with an anastomosis height between 2 and 8 cm from the anal verge and a planned diverting stoma qualifying for participating in the SEVTAR study. Two centimeters are required to secure the sponge and cover the anastomosis and no additional air is sucked. If the anastomosis would be higher than 8 cm, it might not be reached by the sponge and, commonly, a diverting stoma is not required. The diverting stoma is required when the sponge is unlikely to be blocked by stool.

Patients were informed orally and in writing, and if they were willing to participate, they had to sign a declaration of consent before inclusion. After this, randomization will take place.

### Randomization

After the written consent is signed, the participating hospital will contact the study center transferring a form asking for randomization. For each participating hospital, 10 lots with equal number for treatment and control lots are objected. One of these lots is drawn every time a participating center requests for randomization. The result of randomization is transferred back until 8 a.m. the next morning the latest.

### Surgery and EVT

Surgery itself is performed as usual in the participating center. There are no rules given by the study. After finishing the operation, a sponge should be placed in the treatment group endoscopically *via* rectoscopy or palpatory method with the center of the sponge lying on the intraluminal anastomosis. The sponge parameters are 100–125 mmHg suction with medium intensity and continuous suction. After 5 days, the suction is turned off and the sponge is removed without checking the anastomosis generally.

All other treatments are done as usual in the participating center. Further treatment after the operation is documented in a standardized documentation form including questions of searching for AL and potential treatment of AL.

There will be a check for anastomotic leakage after 30 days. A questionnaire will be completed *via* telephone or during a planned check up in the office. Indicators for AL include secretion of pus or blood transanally, pain, fever, etc. In all of these cases, the participating hospital takes action to search for AL. Primary end point is the rate of anastomotic leakages. Secondary end points are the quality of TME and the method of treating AL.

### Sample size and statistical analysis

The sample size was calculated with G-Power 3.1. The study will have 60% power to detect a difference of 10%, at a one-sided alpha significance level of 5%, assuming an anastomosis leakage rate of 10%–15%.

Altogether, 362 patients have to be analyzed.

Statistical analysis will be performed using Statistical Package for Social Science (SPSS) version 23.0. All data are regularly transferred from the documentation forms to the database. Statistical analysis will be done using Pearson's *χ*^2^ test (Fisher's exact test).

### Abortion of the study

After including 50 patients in each block, statistical analysis searching for side effects will be done. In case of major complications of EVT, or significant differences between groups, the study will be aborted.

## Limited results

Until November 11, 2022, 73 patients have been randomized in the study. Twelve patients had to be excluded for one the following reasons: withdrawal of consent before the operation or intraoperative change of treatment (no anastomosis or no protective stoma).

Results are available for 47 patients. So far, there have been four cases of AL in the control group and two in the EVT group without EVT-related complications.

**FLOWCHART 1:**
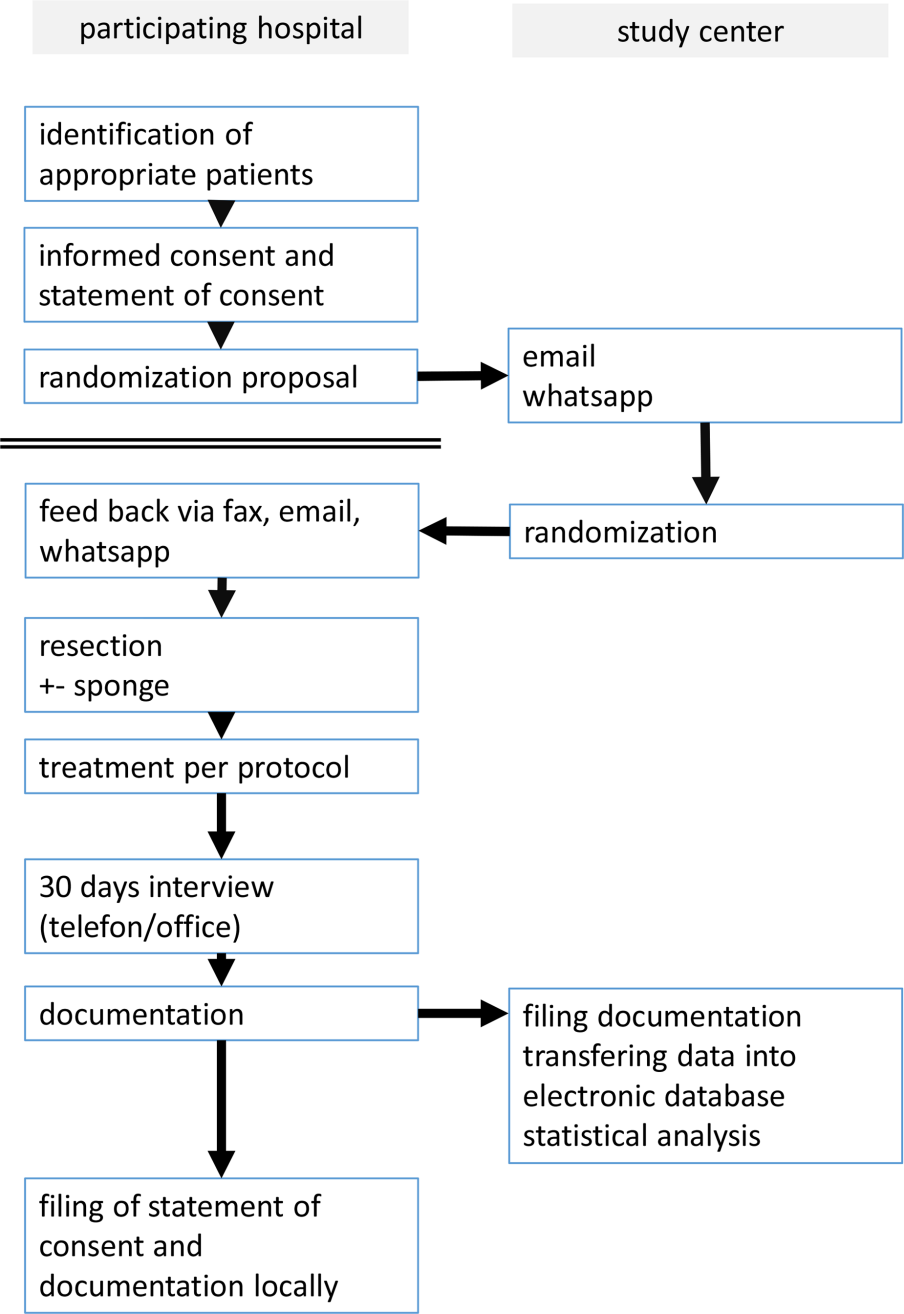
The flowchart shows the process from recruiting the patient to filing the documentation sheets.

**FLOWCHART 2:**
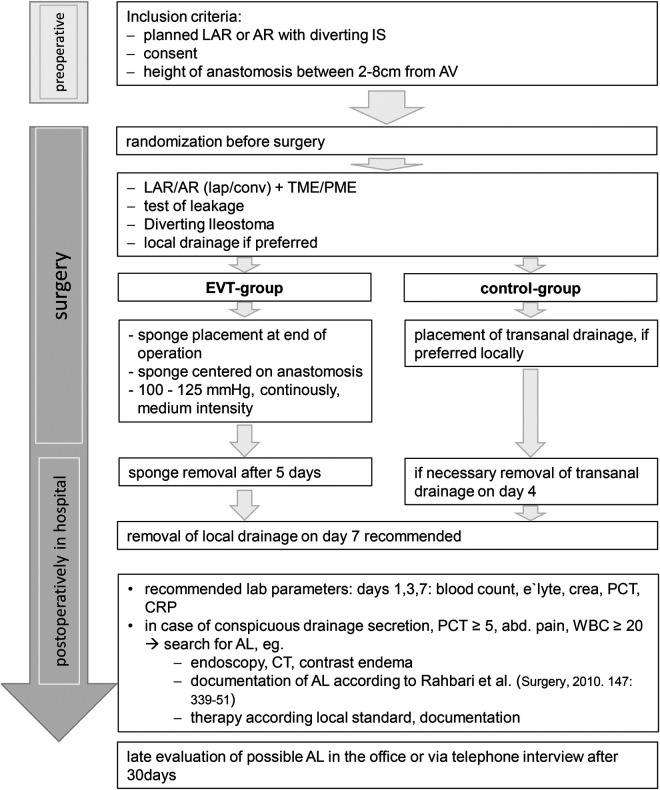
The flowchart shows the SEVTAR study.

## Discussion

If the hypothesis proves to be true, anastomosis leakage could be reduced significantly by placing a vacuum sponge over the anastomosis for 5 days. This would be a very easy method to reduce AL, probably without side effects.

Any hospital interested in participating in the SEVTAR study is encouraged to contact the corresponding author.

## References

[1] Sato T Ozawa H Hatate K Onosato W Naito M Nakamura T A phase II trial of neoadjuvant preoperative chemoradiotherapy with S1 plus irinotecan and radiation in patients with locally advanced rectal cancer: clinical feasibility and response rate. Int J Radiat Oncol Biol Phys. (2011) 79:677–83. 10.1016/j.ijrobp.2009.11.007 21035953

[2] Horisberger K Hofheinz RD Palma P Volkert AK Rothenhoefer S Wenz F Tumor response to neoadjuvant chemoradiation in rectal cancer: predictor for surgical morbidity? Int J Colorectal Dis. (2008) 23:257–64. 10.1007/s00384-007-0408-6 18071720

[3] Ulrich AB Seiler C Rahbari N Weitz J Büchler MW. Diverting stoma after low anterior resection: more arguments in favor. Dis Colon Rectum. (2009) 52:412–8. 10.1007/DCR.0b013e318197e1b1 19333040

[4] Ulrich A Weitz J Büchler MW. Protective stoma after deep anterior rectal resection: pro. Chirurg. (2010) 81(962):964–7. 10.1007/s00104-010-1928-0 20859606

[5] Weidenhagen R Gruetzner KU Wiecken T Spelsberg F Jauch KW. Endoscopic vacuum-assisted closure of anastomotic leakage following anterior resection of the rectum: a new method. Surg Endosc. (2008) 22:1818–25. 10.1007/s00464-007-9706-x 18095024

[6] Kühn F Janisch F Schwandner F Gock M Wedermann N Witte M Comparison between endoscopic vacuum therapy and conventional treatment for leakage after rectal resection. World J Surg. (2020) 44:1277–82. 10.1007/s00268-019-05349-5 31965274

[7] Kühn F Schardey J Wirth U Schiergens T Crispin A Beger N Endoscopic vacuum therapy for the treatment of colorectal leaks—a systematic review and meta-analysis. Int J Colorectal Dis. (2022) 37:283–92. 10.1007/s00384-021-04066-7 34817647 PMC8803669

[8] Talboom K Greijdanus NG van Workum F Ubels S Rosman C for TENTACLE-Rectum working group. International expert opinion on optimal treatment of anastomotic leakage after rectal cancer resection: a case-vignette study. Int J Colorectal Dis. (2022) 37:2049–59. 10.1007/s00384-022-04240-5 36002748 PMC9436864

